# Resources and applications of public biomedical data

**DOI:** 10.3389/fbinf.2026.1782956

**Published:** 2026-05-22

**Authors:** Mingrui Liu, Zelin Ye, Haiyu Liu, Pengzhen Ma, Huaxin Pang, Yaning Li, Qihao Wang, Yikang Shen, Xiaoxia Xie, Yufeng Zhao

**Affiliations:** 1 Data Center of Traditional Chinese Medicine, China Academy of Chinese Medical Sciences, Beijing, China; 2 Department of Infection, Guang’anmen Hospital, China Academy of Chinese Medical Sciences, Beijing, China

**Keywords:** application, big data, data mining, guideline, public biomedical database

## Abstract

This review provides a representative overview of public biomedical databases and their use in biomedical research. These resources are categorized into four major types according to their dominant data content: public health databases, clinical databases, comprehensive cohort databases, and omics databases. For each category, we briefly summarize their main characteristics and access pathways. At the application level, we outline their major uses in population health monitoring, clinical research, predictive modeling, and biomarker discovery. At the methodological level, we summarize two complementary research strategies commonly used with these resources, namely, hypothesis-driven and data-driven research. We further discuss the main challenges in using public biomedical databases and emphasize broad principles for rigorous and appropriate use. Overall, public biomedical databases have become an important infrastructure for modern research. This review aims to provide a reference framework for researchers to more efficiently and reliably utilize these resources for scientific exploration and clinical translation.

## 1 Introduction

With the continuous progress of computer software, hardware and internet technology, the scale and generation speed of global data are showing an unprecedented growth trend (Wu et al., 2021). Big data has become an important feature of the digital age and has had a profound impact in many fields such as healthcare. In the past few decades, the volume and complexity of biomedical data have accelerated (Schlick et al., 2018), driven mainly by the informatization of electronic health records (Coughlin et al., 2018), the popularization of medical imaging and sensing devices (Van Horn and Toga, 2014), the continuous iteration of high-throughput omics technology (Schmidt and Hildebrandt, 2017), and the normalization of cross institutional collaborative research (Smith et al., 2020). Big data is usually summarized by 4 Vs. characteristics, namely, Volume, Variety, Velocity, and Veracity (Bellazzi, 2014). In the field of biomedical science, these features are particularly prominent: they not only cover structured and unstructured text, image, and signal data, but also exhibit the characteristics of large data generation scale and high update frequency (Dinov, 2016). In addition, biomedical data is usually collected based on specific research or medical protocols, with a relatively standardized structure, but at the same time, it is difficult to obtain and often accompanied by source heterogeneity and significant differences in quality (Wang and Krishnan, 2014; Scruggs et al., 2015). The dual challenges of scale and complexity pose higher demands on biomedical research. In this context, with the continuous advancement of information technology, biomedical databases are constantly developing and improving, and their open sharing trend is gradually emerging, becoming an important infrastructure for promoting scientific research cooperation and knowledge discovery. The development of open biomedical databases represents transformative progress. By managing and disseminating large-scale datasets through structured platforms, these resources greatly reduce barriers to data access and create opportunities for interdisciplinary researchers to conduct high impact research without the high cost of collecting raw data.

This review aims to provide a practical overview of representative public biomedical databases for researchers seeking to use these resources in biomedical studies. To identify databases for inclusion, we drew on targeted Web of Science searches, official database websites and portals, database descriptor papers, methodological reviews, and cross-referencing of key resources. Search terms included general resource-related terms (e.g., database, registry, repository, portal, biobank, and cohort) together with category-specific terms related to public health, clinical, and omics data. The literature search and website checks were updated through August 26, 2025. Resources included in this review were selected based on the following considerations: (i) they had an official website or portal and sufficient documentation to allow verification of their scope, content, and access conditions; and (ii) they were either publicly accessible or available through a clearly defined research access pathway, and they were documented in database descriptor papers, methodological reviews, or other citable biomedical sources. The databases included in this review were selected to provide a representative, rather than exhaustive, overview of major public biomedical databases across different data types and research contexts, thereby offering a structured reference for their appropriate selection and use in biomedical research.

## 2 Classification and overview of public biomedical databases

In this review, we classify publicly available biomedical databases into four categories—public health databases, clinical databases, comprehensive cohort databases, and omics databases—based on their dominant data type and primary research context, in order to provide a clearer framework for database selection.

### 2.1 Public health databases

Public health databases refer to resources that primarily capture population health status and related behavioral, environmental, and social factors. The data contained in these databases are mainly derived from health surveys, surveillance systems, and disease-burden estimation resources.

Table 1 provides an overview of representative public health databases, including their scope, access conditions, and technical requirements for data access. In practice, these databases are most useful for epidemiologic and public health analyses at the population level. For the same research question, different public health databases can provide complementary evidence at different analytic levels: nationally representative surveys such as National Health and Nutrition Examination Survey (NHANES) and Korea National Health and Nutrition Examination Survey (KNHANES) can be used to estimate the prevalence and distribution of key health indicators across population groups; studies such as The Health and Retirement Study (HRS) and English Longitudinal Study of Ageing (ELSA) can be used to assess whether similar patterns are observed across countries; and disease-burden or surveillance resources such as Global Burden of Disease (GBD) can contextualize these findings in terms of broader population burden and temporal trends. Compared with clinical and comprehensive cohort databases, public health databases generally offer broader representativeness and more standardized measurement of non-clinical factors, but provide less detailed information on clinical management and disease severity. Their data are often distributed in structured formats and are relatively easy to access, typically without requiring specialized computational expertise for downloading or initial data retrieval, which lowers the practical barrier to access for researchers, including clinicians.

**TABLE 1:** Overview of representative public health databases.

Database	URL	Notes	Access permissions	Technical requirements for data access	References
National Health and Nutrition Examination Survey (NHANES)	https://www.cdc.gov/nchs/nhanes/index.html	NHANES is a continuous cross-sectional program assessing the health and nutritional status of the noninstitutionalized US population. Since 1999, it has enrolled approximately 5,000 participants annually and released data in 2-year cycles. It provides nationally representative data from household interviews, physical examinations, and laboratory testing, covering demographics, diet, health behaviors, anthropometric measures, and clinical biomarkers	Free	No specialized technical expertise required for data access	(Ahluwalia et al., 2016) (Zipf et al., 2013)
China Health and Nutrition Survey (CHNS)	https://chns.cpc.unc.edu/	CHNS is a longitudinal cohort study designed to examine health and nutrition changes during China’s rapid socioeconomic transition. Across nine survey waves from 1989 to 2011, it collected longitudinal data from 12 provinces with substantial geographic and developmental diversity. Using a multistage stratified cluster sampling design, CHNS provides data at the individual, household, and community levels, covering demographics, dietary intake, anthropometric measures, health behaviors, healthcare utilization, and community infrastructure	Community-level data require application and approval	No specialized technical expertise required for data access	Popkin et al. (2010) Zhang et al. (2014)
South Korea National Health and Nutrition Examination Survey (KNHANES)	https://knhanes.kdca.go.kr/knhanes/main.do	KNHANES is a nationally representative cross-sectional survey in South Korea that has been conducted since 1998, with annual surveys organized into 3-year rolling cycles since 2007. KNHANES collects data through health interviews, physical examinations, and dietary assessments, covering anthropometric measurements, health examination, health behaviors, and nutrient intake	Free	No specialized technical expertise required for data access	Lee et al. (2025) Oh et al. (2021) Kweon et al. (2014)
Chinese Longitudinal Healthy Longevity Survey (CLHLS)	https://opendata.pku.edu.cn/dataverse/CHADS	CLHLS is a prospective cohort study of adults aged 65 years and older in China, with additional data collected from their adult children. Since 1998, it has completed eight follow-up waves across 23 provinces, producing both cross-sectional and longitudinal data. CLHLS covers demographics, health status, cognitive function, psychological characteristics, activities of daily living, healthcare utilization, and mortality, and also includes intergenerational follow-up data from parent-child pairs, biomedical examination data from selected longevity areas, and matched community-level data on socioeconomic and environmental factors	Restricted datasets require application and approval	No specialized technical expertise required for data access	Zeng (2012)
Health and Retirement Study (HRS)	https://hrs.isr.umich.edu/	HRS, ELSA, MHAS, SHARE, JSTAR, and CHARLS are part of a broader family of nationally representative longitudinal studies on ageing and retirement that largely follow the conceptual framework of the US HRS. These surveys typically enroll adults aged 45 or 50 years and older and collect broadly comparable data on health status, chronic diseases, cognitive function, mental health, economic status (including income, assets, and pensions), employment and retirement history, healthcare utilization, family structure, and death-related information. Similar datasets have also been established in other settings, including KLoSA (Korea), LASI (India), TILDA (Ireland), ELSI-BRASIL (Brazil), NICOLA (Northern Ireland), HART (Thailand), and IFLS (Indonesia) To support cross-national comparisons, harmonized datasets have been developed. These datasets convert the original study-specific files into user-friendly formats with consistently defined and clearly labeled variables. They follow the conventions first established in the RAND HRS Longitudinal File, enabling summary analyses across countries and standardized longitudinal tracking within each study. Each harmonized dataset integrates all available survey waves and is accompanied by detailed documentation and codebooks, which are freely accessible through the Global Aging Data Portal (https://g2aging.org/home)	Free	No specialized technical expertise required for data access	Sonnega et al. (2014) Steptoe et al. (2013) Wong et al. (2023) Börsch-Supan et al. (2013) Ichimura et al. (2009) Zhao et al. (2014)
English Longitudinal Study of Ageing (ELSA)	https://www.elsa-project.ac.uk/				
Mexican Health and Aging Study (MHAS)	https://www.mhasweb.org/Home/index.aspx				
Survey of Health, Ageing and Retirement in Europe (SHARE)	https://share-eric.eu/				
Japanese Study of Aging and Retirement (JSTAR)	https://www.rieti.go.jp/en/projects/jstar/index.html				
China Health and Retirement Longitudinal Study (CHARLS)	https://charls.pku.edu.cn/				
Midlife in the United States (MIDUS)	https://midus.wisc.edu/	MIDUS is a nationally representative longitudinal cohort study of American adults designed to investigate the biopsychosocial determinants of health and wellbeing. The baseline survey was conducted in 1995–1996 among adults aged 25–74 years, with main follow-up survey waves conducted in 2004–2006 and 2013–2014. Ancillary components, including daily diary, cognitive, biomarker, neuroscience, and genomics assessments, were conducted in subsamples over extended periods	Restricted repositories and requested data require application and approval	No specialized technical expertise required for data access	Radler and Ryff (2010) Radler (2014)
Millennium Cohort Study (MCS)	https://cls.ucl.ac.uk/cls-studies/millennium-cohort-study/	MCS is a prospective longitudinal cohort study that tracks the development of children born in the United Kingdom between 2000 and 2002. This study collects rich multi-source data from cohort members and their parents at multiple time points, covering physical health, cognitive and emotional development, educational performance, family background, and parenting practices. MCS data is associated with birth registration records, hospital records, education records, and geographic records, supporting longitudinal studies on early determinants of health and development	Sensitive data require application and approval	No specialized technical expertise required for data access	Connelly and Platt (2014) Hockley et al. (2008)
Global Burden of Disease (GBD)	https://www.healthdata.org/research-analysis/gbd	GBD database provides comprehensive and standardized estimates of morbidity, mortality and risk factors in 204 countries and regions since 1990. GBD integrates data from health surveys, hospital records, vital signs, and systematic reviews, covering over 371 diseases and injuries, as well as thousands of sequelae. GBD follows five core principles: generating the best estimate with quantitative uncertainty, comprehensively accounting for diseases and risks, ensuring comparability across time and place, incorporating mortality and non-fatal health outcomes, and verifying results through expert review and internal consistency checks	Free	No specialized technical expertise required for data access	Murray (2022)
CDC WONDER	https://wonder.cdc.gov/	The CDC WONDER system is a centralized portal for accessing various public health datasets in the US. It combines mortality and birth statistics, cancer incidence rate and mortality, reportable infectious disease surveillance and environmental health data, and demographic information	Free	No specialized technical expertise required for data access	Friede et al. (1993)

### 2.2 Clinical databases

Clinical databases refer to resources that primarily capture patient-level data generated during routine care in real-world healthcare settings. The data contained in these databases are mainly derived from disease registries, electronic health records (EHRs), and other time-stamped clinical records.

Table 2 summarizes representative clinical databases, with emphasis on their scope, access conditions, and technical requirements for data access. Clinical databases are most useful when the research question requires detailed characterization of diagnoses, treatments, disease severity, short-term outcomes, or dynamic physiological changes during clinical care. This category is internally heterogeneous, and resource selection should be guided by the research question. For example, even within cancer registries, database choice depends on the analytic aim: Surveillance, Epidemiology, and End Results (SEER) is population-based and better suited to questions requiring broader population representation, whereas National Cancer Database (NCDB) is hospital-based and often provides larger case numbers. More broadly, registry-based databases are better suited for comparing outcomes across patient groups and identifying factors associated with prognosis, whereas EHR-based datasets are better suited for studying in-hospital disease progression, treatment processes, and short-term outcomes. Compared with public health databases, clinical databases generally offer greater clinical detail, but their representativeness is often constrained by healthcare setting, referral patterns, or institutional scope. Access and technical burden also vary across this category. Registry-based resources are often available as relatively structured files, whereas EHR-based datasets usually require more complex extraction and preprocessing, sometimes involving relational database queries such as SQL; waveform and imaging data often require more specialized technical expertise for data processing and analysis.

**TABLE 2:** Overview of representative clinical databases.

Database	URL	Notes	Access permissions	Technical requirements for data access	References
Surveillance, Epidemiology, and End Results (SEER)	https://seer.cancer.gov/	SEER is a population-based cancer registry program in the US. The dataset described here is the Incidence – SEER Research Limited-Field Data, 22 Registries, November 2023 Submission, covering tumors diagnosed from 2000 to 2021. It contains 17,666,410 tumor-level records and represents approximately 47.9% of the US population based on the 2020 census. SEER provides structured data on patient demographics, socioeconomic and geographic characteristics, tumor morphology and location, diagnostic stage, initial treatment methods, as well as survival and mortality follow-up	Registration and data use agreement required	No specialized technical expertise required for data access	Park et al. (2012) Che et al. (2023)
National Cancer Database (NCDB)	https://www.facs.org/quality-programs/cancer-programs/national-cancer-database/	NCDB is a hospital-based cancer registry that gathers data from nearly 1,500 hospitals in the US, covering over 72% of newly diagnosed cancer patients, including 75 different cancer-related diseases. By 2016, NCDB had amassed more than 34 million records of patients with cancer. The data is extracted from medical records by certified tumor registrants, including patient demographic data, tumor characteristics, comorbidities, and treatment during the first course of care defined before disease progression or recurrence	Research access is provided through PUFs for investigators at CoC-accredited programs upon application and approval	No specialized technical expertise required for data access	Mallin et al. (2019) Boffa et al. (2017) McCabe (2019)
The Cancer Imaging Archive (TCIA)	https://www.cancerimagingarchive.net/	TCIA is a public database supported by the National Cancer Institute in the US, aimed at promoting cancer imaging research and enhancing reproducibility. It contains a large and constantly growing selection of computed tomography (CT), magnetic resonance imaging (MRI), and positron emission tomography (PET) images for de identification, organized by tumor type. Many collections also include supplementary data such as clinical records, image annotations, pathological sections, radiation therapy plans, and links to genomic resources such as the Cancer Genome Atlas (TCGA)	Free	No specialized technical expertise required for data access	Prior et al. (2017) Clark et al. (2013)
Medical Information Mart for Intensive Care (MIMIC)	https://mimic.mit.edu/	MIMIC is a single-center, de-identified critical care and emergency department database derived from Beth Israel Deaconess Medical Center. It integrates multiple data types, including clinical records from the intensive care units (ICU) information systems and hospital archives, high-resolution physiological data such as waveforms and time series of vital signs and alarms from bedside monitors, and mortality data. Across its major generations, MIMIC has evolved from the original MIMIC database covering 1994–1996, through MIMIC-II and MIMIC-III, to MIMIC-IV. The dataset described here is MIMIC-IV v3.1, released in October 2024, which covers emergency department or ICU admissions from 2008 to 2022. It contains 364,627 unique individuals, 546,028 hospitalizations, and 94,458 ICU stays	Credentialed access; data use agreement and human-subjects training required	Basic SQL or database-handling skills required	Mark (2016) Rogers et al. (2021)
eICU Collaborative Research Database (eICU-CRD)	https://eicu-crd.mit.edu/	The eICU-CRD is a multicenter, de-identified intensive care dataset that includes data from more than 200,000 ICU admissions across the US in 2014 and 2015. The dataset includes patient demographics, bedside vital signs, laboratory measurements, APACHE IV severity scores, diagnoses, treatments, medications including infusions, nursing plans, nursing assessments, and other clinical documentation. Based on the MIMIC project, eICU-CRD extends from a single-center dataset to a multicenter resource compiled from numerous hospitals across the US.	Credentialed access; data use agreement and human-subjects training required	Basic SQL or database-handling skills required	Pollard et al. (2018)
National Sleep Research Resource (NSRR)	https://sleepdata.org/	NSRR is a centralized repository for sleep and circadian research that curates de-identified polysomnography, actigraphy, questionnaire-based, and associated clinical data from multiple cohorts, clinical trials, and other data sources. As of 1 May 2026, the NSRR portal reported 54,119 individuals represented. Because NSRR aggregates datasets from multiple independent studies and incorporates resources over time, it has no single platform-wide data-collection period or follow-up cutoff. NSRR uses Sleep Common Data Elements that comprise more than 900 core sleep terms covering demographic information, anthropometric parameters, physiologic measurements, medical history, sleep-study data, sleep symptoms, polysomnography events, laboratory data, and neurocognitive testing results	Registration and dataset-specific data use agreement required	No specialized technical expertise required for standard data access	Zhang et al. (2018)
Alzheimer’s Disease Neuroimaging Initiative (ADNI)	https://adni.loni.usc.edu/	ADNI is a large-scale, multi-site, longitudinal study aimed at standardizing and validating biomarkers for the diagnosis, progression monitoring, and treatment trials of Alzheimer’s disease (AD). ADNI integrates multimodal data, including structural and functional MRI, PET, clinical and neuropsychological assessments, biological sample analysis, and genetic data	Registration and approved data use application required	No specialized technical expertise required for standard data access	Hendrix et al. (2015) Toga et al. (2024) Weiner and Veitch (2015)
Open Access Series of Imaging Studies (OASIS)	https://sites.wustl.edu/oasisbrains/	OASIS provides publicly available neuroimaging datasets to advance research on normal aging and AD. Its published content includes OASIS-1 (cross-sectional MRI of the entire adult lifespan), OASIS-2 (longitudinal MRI of elderly individuals with and without dementia), OASIS-3 (longitudinal multimodal imaging, cognitive and biomarker data), and OASIS-4 (clinical cohort with neuroimaging and biomarker assessment)	Registration and data use agreement required	No specialized technical expertise required for standard data access	Marcus et al. (2007) LaMontagne et al. (2019)
DeepLesion	https://nihcc.app.box.com/v/DeepLesion	The DeepLesion database is constructed by mining lesion annotations from large clinical Picture Archiving and Communication System. It contains 32,735 annotated lesions from 32,120 CT slices from 10,594 studies of 4,427 unique patients. Unlike datasets that focus on a single lesion type, DeepLesion includes various pathologies of multiple body parts, making it a valuable resource for developing universal lesion detection algorithms. Each lesion is labeled with a bounding box from a conventional radiologist’s bookmark, and the dataset also provides relevant measurements such as lesion diameter	Free	No specialized technical expertise required for standard data access	Yan et al. (2018)

### 2.3 Comprehensive cohort databases

Comprehensive cohort databases refer to longitudinal resources that primarily capture multidimensional individual-level data collected from the same participants over time. The data contained in these databases are mainly derived from baseline assessments, repeated follow-up, and linkage to subsequent health records, and in some cohorts also include imaging or omics data.

Table 3 outlines representative comprehensive cohort databases. Comprehensive cohort databases are particularly useful when the research question requires integration of multidimensional data from the same individuals and assessment of their associations with subsequent health outcomes over time. When variables and outcome definitions are sufficiently comparable across cohorts, analyses in different cohorts can also help assess whether findings are consistent across populations. Within this category, database selection depends on both data breadth and the access framework. For example, compared with China Kadoorie Biobank (CKB), United Kingdom Biobank (UKB) provides a broader range of data types, including more extensive genetic, imaging, and other omics resources, making it better suited for large-scale data-driven analyses such as phenome-wide association studies and exposure-wide association studies, whereas CKB is more oriented toward hypothesis-driven analyses under its current data-sharing framework. Compared with public health and clinical databases, comprehensive cohort databases generally provide more integrated longitudinal data across exposures, phenotypes, outcomes, and, in some cohorts, imaging or omics profiles, allowing multiple data dimensions to be related to later health outcomes. Access and analytic burden also tend to be higher in this category, often involving formal application, governance review, and fee-based access. Some resources, such as UKB, require analysis within secure cloud-based or other controlled research environments rather than through simple local download, which increases the technical demands on researchers.

**TABLE 3:** Overview of representative comprehensive cohort databases.

Database	URL	Notes	Access permissions	Technical requirements for data access	References
United Kingdom Biobank (UKB)	https://www.ukbiobank.ac.uk/	UKB is a large-scale prospective cohort that recruited approximately 500,000 participants aged 40–69 in the United Kingdom between 2006 and 2010. It provides baseline information for identification through questionnaire surveys, physical examinations, and biological samples (blood, urine, saliva), supplemented by genotyping, whole exome, and whole genome sequencing data. Long term follow-up is achieved through repeated questionnaire surveys and extensive connections with national electronic health records, hospitalization, cancer, and death registration. In addition, UKB provides multimodal imaging data, including brain, heart, abdominal, carotid, and musculoskeletal scans, as well as physiological monitoring. Recently, the UKB incorporated COVID-19 data, including hospitalization, death, test records and specialized sub studies	Fee-based; application, approval, and Material Transfer Agreement required	Cloud-based access via UKB-RAP; platform-specific computational skills required	Trehearne (2016) Allen et al. (2024) Feng et al. (2023)
China Kadoorie Biobank (CKB)	https://www.ckbiobank.org/	CKB is one of the largest population-based prospective cohorts in Asia. It recruited 512,891 adults aged 30–79 years from 10 geographically and socioeconomically diverse regions in China, covering both urban and rural populations. Baseline data collection included questionnaires on lifestyle, medical history, and socioeconomic status, together with physical measurements and blood sampling. Follow-up was conducted through linkage to mortality, disease incidence, and hospitalization records, supplemented by regular reexamination. Data currently available to researchers include baseline and repeated survey data, health outcomes through 2017, and subsets of biochemical, metabolomic, and proteomic data, while additional omics resources are expected to be released in the future	Free for applicants from mainland China and Hong Kong, fee-based for researchers elsewhere; application and approval required	No specialized technical expertise required for standard data access	Chen et al. (2011)

### 2.4 Omics databases

Omics databases refer to resources that primarily capture high-dimensional molecular data, including genomic, transcriptomic, epigenomic, proteomic, metabolomic, and related molecular data. The data contained in these databases are mainly derived from high-throughput molecular profiling experiments.

Table 4 summarizes representative public omics databases and resources. Within this category, resources can be broadly grouped into raw sequencing archives (e.g., Sequence Read Archive and European Nucleotide Archive), general repositories (e.g., Gene Expression Omnibus and ArrayExpress), disease-focused integrative multi-omics resources (e.g., The Cancer Genome Atlas Program), reference and annotation resources (e.g., The 1000 Genomes Project, The Encyclopedia of DNA Elements, and Roadmap Epigenomics), and protein- or metabolite-level reference resources (e.g., Human Protein Atlas and Human Metabolome Database). Accordingly, database selection should be guided by whether the study requires primary sequence data, independent validation, disease-focused integrative analysis, functional annotation and biological context, or downstream protein- or metabolite-level interpretation. Omics databases are particularly useful when the research question requires direct molecular evidence to characterize biological processes or disease mechanisms. They can also complement findings from other public biomedical databases by supporting mechanistic interpretation and candidate biomarker or therapeutic target discovery. Although many omics resources are relatively straightforward to access and download, the analytical burden is often substantial, as preprocessing, normalization, batch correction, feature annotation, and multi-platform integration typically require specialized bioinformatics expertise.

**TABLE 4:** Overview of representative omics databases and resources.

Database	URL	Notes	Access permissions	Technical requirements for data access	References
Sequence Read Archive (SRA)	https://www.ncbi.nlm.nih.gov/sra	SRA is a public archive of high-throughput sequencing data that stores raw sequencing data and alignment information from a wide range of studies, including those across all branches of life as well as metagenomic and environmental surveys. As one of the largest publicly available repositories of sequencing data, it provides an important resource for data reuse, reproducibility, and secondary analysis	Free for public data; protected human data require controlled-access authorization	Basic toolkit-based or cloud-based access skills may be required	Katz et al. (2022)
European Nucleotide Archive (ENA)	https://www.ebi.ac.uk/ena/browser/home	ENA is an open nucleotide sequence archive maintained by EMBL-EBI that provides a comprehensive record of global sequencing information, covering raw sequencing data, sequence assembly information, and functional annotation. It also captures contextual metadata related to samples, experimental workflows, and sequencing projects, thereby supporting data submission, retrieval, integration, and reuse across diverse sequencing-based studies	Free	Bulk download may involve API or large-scale file transfer tools	Burgin et al. (2023)
Gene Expression Omnibus (GEO)	https://www.ncbi.nlm.nih.gov/geo/	GEO is one of the largest public repositories for high-throughput molecular abundance data. It archives transcriptomic, genomic, and proteomic profiles generated by both array- and sequencing-based technologies, together with associated metadata describing samples, platforms, and study design. Its records are organized into three core entities—Platform, Sample, and Series—thereby supporting data submission, retrieval, cross-study comparison, and secondary analysis	Free	Bulk download may involve FTP or programmatic access	Edgar et al. (2002)
ArrayExpress	https://www.ebi.ac.uk/biostudies/arrayexpress	ArrayExpress is a public functional genomics repository that primarily archives transcriptomic data, especially microarray-based gene expression profiles, together with associated experimental metadata. It also includes other array-based functional genomics datasets, such as comparative genomics and ChIP-chip experiments. Since the retirement of the standalone ArrayExpress interface in 2022, ArrayExpress data are now accessible through the BioStudies ArrayExpress collection at EMBL-EBI.	Free	Bulk download may involve FTP, Aspera, or API-based access	Brazma et al. (2003)
The Cancer Genome Atlas Program (TCGA)	https://www.cancer.gov/ccg/research/genome-sequencing/tcga	TCGA is a landmark cancer genomics program jointly launched by the National Cancer Institute and the National Human Genome Research Institute in 2006. It characterized over 20,000 primary tumor and matched normal samples across 33 cancer types, generating more than 2.5 PB of linked clinical, genomic, epigenomic, transcriptomic, and proteomic data	Open access for public data; controlled-access authorization required for protected data	Bulk or controlled-data download may require use of the GDC Data Transfer Tool or API.	Wang et al. (2016)
1000 Genomes Project	https://www.internationalgenome.org/home	The 1000 Genomes Project Phase 3 final dataset included genomic data from 2,504 individuals across 26 populations worldwide. Completed in 2015, the project used low-coverage whole-genome sequencing, deep exome sequencing, and dense microarray genotyping to characterize common and rare human genetic variation. This resource provides a global reference for population genetics and supports studies linking genetic variation to human health and disease	Free	Bulk download may involve FTP, HTTP, Aspera, or Globus	The 1000 Genomes Project Consortium et al. (2015)
Encyclopedia of DNA Elements (ENCODE)	https://www.encodeproject.org/	The ENCODE project systematically identified functional elements in the human and mouse genomes. It provides comprehensive functional genomics resources, including epigenomics and transcriptome data for different human and mouse cell types	Free	Bulk download may involve REST API or metadata-based batch retrieval	Luo et al. (2020)
Roadmap Epigenomics	https://egg2.wustl.edu/roadmap/web_portal/index.html	The NIH Roadmap Epigenomics Mapping Consortium has created a genome-wide map of histone modifications, chromatin accessibility, DNA methylation, and mRNA expression across different human cell types and tissues. In the 2015 integrative analysis, the consortium analyzed 111 Roadmap reference human epigenomes; together with additional ENCODE reference epigenomes, these data provided a unified resource for functional and translational research	Free	Bulk retrieval may involve portal-based archives or integrated access through ENCODE/GEO resources	Roadmap et al. (2015)
Functional Annotation of the Mammalian Genome (FANTOM)	https://fantom.gsc.riken.jp/	The FANTOM project is an international consortium that is gradually advancing transcriptome research. Its resources are mainly concentrated in the transcriptome, including promoters, enhancers, long non-coding RNAs, microRNAs, and a comprehensive map of transcriptional regulatory networks across multiple species	Free	Bulk download or subset extraction may involve FTP-based download or use of FANTOM tools such as TET.	Abugessaisa et al. (2021)
The Genotype-Tissue Expression (GTEx)	https://www.gtexportal.org/home/	The GTEx project integrates genomics and transcriptomics by combining whole genome sequencing with RNA sequencing across multiple human tissues. It maps the expression of quantitative trait loci, linking genetic variation with tissue-specific gene expression	Open access for public data; controlled-access authorization required for protected genotype and raw sequencing data	Controlled-data access may involve dbGaP/AnVIL authorization and platform-specific workflows	L et al. (2013)
The Human Protein Atlas (HPA)	https://www.proteinatlas.org/	HPA combines transcriptomics, proteomics, and imaging techniques to create protein and gene expression maps across tissues, cell types, subcellular compartments, cancer, blood, and cell lines, including data on protein structure and interactions	Free	Programmatic access or bulk download may involve JSON, XML, or RDF-based retrieval	Digre and Lindskog (2021)
The Human Metabolome Database (HMDB)	https://hmdb.ca/	HMDB is a metabolomics resource that catalogs confirmed human metabolites and their properties, concentrations, related enzymes, pathways, and disease associations, and provides extensive reference spectral data from NMR, LC-MS, and GC-MS.	Free	Bulk download may involve programmatic access or dedicated download tools	Wishart et al. (2022)

## 3 Major applications of public biomedical databases

**FIGURE 1:**
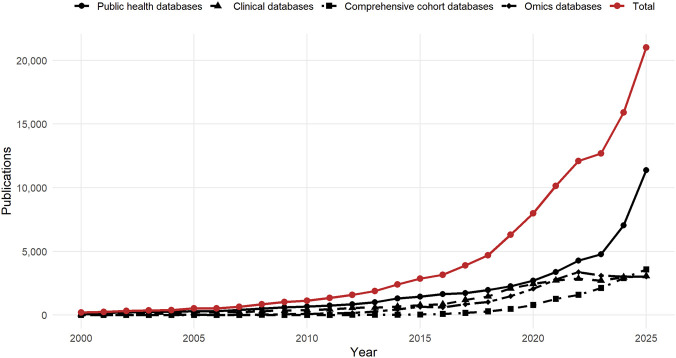
Annual publication trends of Web of Science Core Collection records retrieved by topic searches of representative public biomedical database names listed in Section 2 (Tables 1–4). Records are grouped into four categories: public health databases, clinical databases, comprehensive cohort databases, and omics databases. The Total represents the sum of the annual counts from the four categories. Detailed search strategies, retrieval restrictions, and counting methods are provided in the Supplementary Material.

**FIGURE 2:**
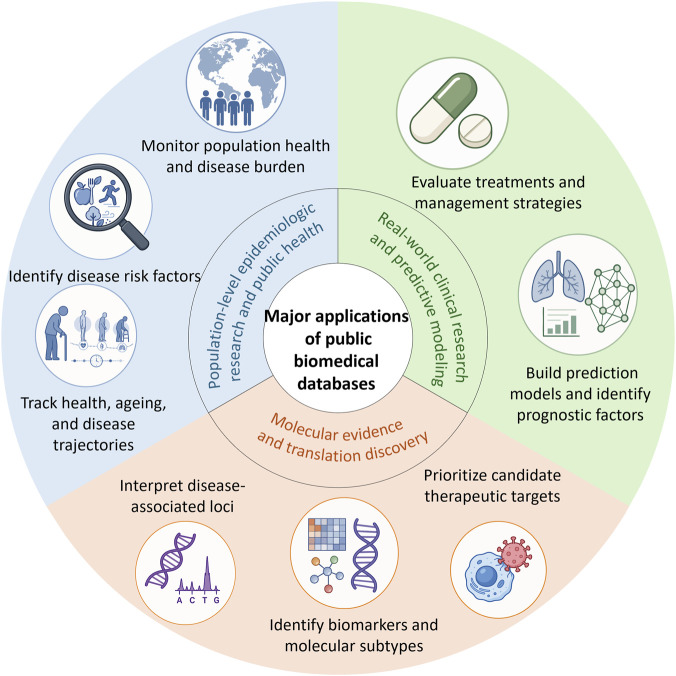
Overview of the application of public biomedical databases.

Public biomedical databases are not just data repositories; they have become indispensable resources for biomedical research. As shown in Figure 1, the annual number of Web of Science Core Collection records retrieved by topic searches of representative public biomedical database names increased markedly from 2000 to 2025, with a clear acceleration after 2015. In this context, we summarize the applications of public biomedical databases mainly in population-level research, clinical research, and molecular discovery (Figure 2).

### 3.1 Population-level epidemiologic research and public health applications

Public biomedical databases are widely used to generate population-level evidence for public health research, prevention, and policy planning. First, standardized resources such as the GBD study support population health surveillance and burden assessment by quantifying incidence, prevalence, mortality, disability, and related burden measures across diseases, populations, regions, and time (GBD, 2016). GBD-based analyses, for example, have been used to assess changes in lower respiratory tract infection mortality and shifts in major etiologies (GBD, 2024), quantify the burden of musculoskeletal disorders (GBD, 2021 Other Musculoskeletal Disorders Collaborators, 2023), and project future gastric cancer burden attributable to high salt intake in different countries (Liu et al., 2025). Second, these databases support the identification of disease risk factors by linking behavioral, environmental, dietary, and socioeconomic factors to health outcomes. For example, databases such as NHANES and harmonized ageing cohorts can be used to examine associations of environmental, behavioral, socioeconomic, and dietary factors with outcomes such as depression, frailty, and cognitive ageing across populations (Parker et al., 2025; Wang et al., 2024; Tian et al., 2022; Lv et al., 2023). Third, longitudinal cohort databases, including harmonized ageing cohorts and comprehensive cohort databases, can be used to examine health, ageing, and disease trajectories over time, including cognitive decline, functional change, pain trajectories, downstream comorbidity patterns, and related changes. For example, harmonized ageing cohorts such as HRS and ELSA can be used to assess how parental education and pain trajectories are associated with later-life cognitive decline, while comprehensive cohort resources such as UKB have been used to map downstream comorbidity patterns among individuals with depression (Han et al., 2021; Luo et al., 2025; He et al., 2024).

### 3.2 Real-world clinical research and predictive modeling

Public biomedical databases are widely used to generate clinically relevant evidence that complements randomized controlled trials, supports risk stratification, and improves understanding of disease course in routine care settings. First, these databases can be used to evaluate the effectiveness and safety of treatments and clinical management strategies in real-world patient populations. For example, registry- and EHR-based resources such as SEER, NCDB, and Medical Information Mart for Intensive Care (MIMIC) have been used to assess the effectiveness and safety of surgical (Xing et al., 2018), radiotherapeutic (Rusthoven et al., 2016), and critical care interventions (Xiong et al., 2023; Hu et al., 2022) in relation to survival and complications in routine clinical practice. Second, public biomedical databases can be used for prognostic assessment and predictive modeling by identifying factors associated with prognosis and developing prediction models for clinically relevant outcomes in patient populations. Such models may also integrate clinical, imaging, and, in some settings, molecular features to improve prediction and support clinically relevant phenotyping or treatment stratification. For example, resources such as Alzheimer’s Disease Neuroimaging Initiative (ADNI), UKB, and The Cancer Genome Atlas Program (TCGA) have been used to assess prognostic biomarkers and develop clinically relevant prediction models for outcomes such as dementia (You et al., 2022; Tang et al., 2024), atrial fibrillation (Segan et al., 2023), and tumor immunophenotypes (Tong et al., 2022; Mazurowski et al., 2013).

### 3.3 Molecular evidence and translational discovery

Public biomedical databases are widely used to generate molecular-level evidence that supports mechanism-oriented research and translational discovery. First, these databases can be used to functionally interpret disease-associated genetic loci by linking them to downstream molecular phenotypes such as gene expression, thereby providing clues to the pathways through which genetic variation may influence disease susceptibility. For example, integrative analyses combining GWAS with GTEx eQTL data have been used to connect migraine risk loci with gene expression and to prioritize candidate genes for further investigation (Ghaffar et al., 2023). Second, public biomedical databases can be used to identify biomarkers and molecular subtypes relevant to diagnosis, prognosis, and treatment stratification by integrating genomics, transcriptomics, proteomics, and related molecular data in complex diseases. For example, multi-omics analyses have been used to identify candidate biomarkers in renal cancer (Qin et al., 2025), reveal gene signatures associated with tumor progression and survival (Saha et al., 2025), and define clinically relevant molecular subtypes such as HER2-enriched profiles across tumor types (Li et al., 2020). Third, these databases can be used to prioritize candidate therapeutic targets and assess their potential effects. For example, analyses integrating genetic association data with molecular evidence have been used to identify candidate therapeutic targets for migraine and ankylosing spondylitis, while further phenome-wide analyses have been used to assess their potential adverse effects and safety-related implications (Zhao et al., 2024; Sun et al., 2024).

Taken together, these applications show the versatility of public biomedical databases in supporting evidence generation across different research contexts.

## 4 Data utilization workflows and research strategies

**FIGURE 3:**
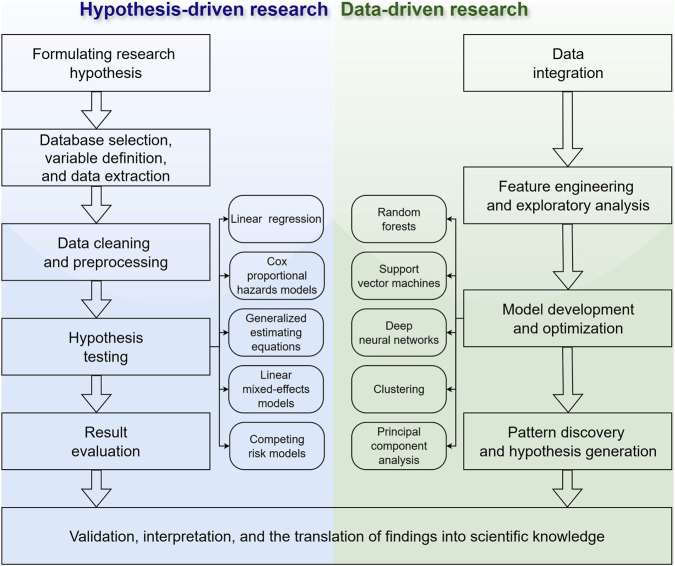
Typical workflow of hypothesis-driven research and data-driven research. The analytical methods listed in the figure are representative examples rather than exhaustive categories, and their placement does not imply that a given method is exclusive to one research strategy. In practice, method selection and interpretation should depend on the research question and analytical objective.

In addition to the main applications mentioned above, it is also crucial to understand how researchers use public biomedical databases to solve scientific problems. In general, two complementary research strategies can be distinguished: hypothesis-driven research and data-driven research (Figure 3). Hypothesis-driven research starts from a predefined scientific, clinical, or biological question and is mainly used to test prior knowledge, evaluate expected associations, or examine biologically plausible mechanisms (Dreisbach and Maki, 2023). Data-driven research, in contrast, starts from complex data and is mainly used to identify patterns, generate candidate signals, or construct predictive models. Accordingly, the distinction between these two strategies lies primarily in the formulation of the research question and the intended interpretation of the results, rather than in the databases used or in the computational complexity of the analytical methods.

Hypothesis-driven and data-driven research should not be equated with a simple division between conventional statistical models and machine-learning methods. Analytical methods should instead be understood as tools that can serve different purposes depending on the research question. Regression-based and survival models may be used to test prespecified associations, but they may also be used for exploratory modeling or preliminary screening of candidate predictors (Shmueli, 2010). Conversely, machine-learning methods may be used in prespecified prediction studies when the target population, outcome, candidate predictors, and performance metrics are defined before model development (Col et al., 2024). In practice, these two strategies are often connected rather than separated. For example, a UKB exposome-wide study of psychotic experiences used agnostic screening to identify candidate nongenetic correlates, followed by replication, multivariable modeling, and Mendelian randomization to assess the robustness and directionality of the associations (Lin et al., 2022). Similarly, a recent UKB study used machine-learning models to predict incident disease cases undiagnosed at recruitment and then leveraged these predictions to augment genetic association and phenome-wide analyses (Garg et al., 2024). These examples show that data-driven discovery, structured evaluation, and biological interpretation can operate as connected stages within the same research cycle.

Database analyses designed for different purposes differ in what conclusions they can support. Confirmatory, exploratory, predictive, and hypothesis-generating analyses should therefore be interpreted according to their intended roles. Researchers should define the intended role of the analysis before drawing conclusions from public biomedical databases, so that the interpretation of findings remains consistent with the study design and analytical objective.

## 5 Challenges and recommended practices in using public biomedical databases

### 5.1 General challenges in using public biomedical databases

The growing availability of public biomedical databases has greatly expanded research opportunities, but availability should not be equated with suitability, validity, or reproducibility. Key general challenges include: (i) database selection driven by convenience or popularity rather than by a clearly defined research question, as reflected in the rapid growth of “formulaic” publications based on large public datasets such as NHANES, many of which follow repetitive designs linking single exposures to specific health outcomes (Science PG, 2025); (ii) causal overinterpretation of associations from public biomedical databases, particularly when temporality cannot be established and confounding cannot be adequately controlled; (iii) overstated performance in predictive or biomarker studies when the same or overlapping data are used for both model development and evaluation; (iv) limited external validity of some published findings due to population bias and non-representative samples; for example, although non-representativeness does not necessarily preclude valid estimation of exposure–disease associations, UKB is not representative of the sampling population, and there is evidence of “healthy volunteer” selection bias (Fry et al., 2017); (v) limited reproducibility when database version, release date, or preprocessing steps are not reported in sufficient detail in published studies, making it difficult for other researchers to reconstruct identical analytic samples; (vi) practical barriers to access and use, including language barriers in country-specific databases, computational expertise and resources needed to download and process large datasets, and restricted-access procedures for clinically sensitive resources that may be opaque, lengthy, or ultimately unsuccessful.

### 5.2 Methodological challenges in using different types of public biomedical databases

Although some challenges are shared, the main methodological risks differ by database type. In public health databases, survey design information is often not properly incorporated into analysis. Public health surveys such as NHANES and KNHANES use complex, stratified, multistage cluster sampling and require the use of sample weights, stratification, and clustering variables; analyses that treat these data as if they were obtained by simple random sampling can yield biased estimates, underestimated standard errors, and overstated significance levels (Kim et al., 2013). In addition, cross-wave or cross-country analyses require careful harmonization, because comparisons may be misleading when survey cycles are combined improperly or when variable definitions and measurement frameworks are not sufficiently comparable across waves or countries.

In clinical databases, several methodological problems are especially common. First, treatment comparisons in clinical databases are often affected by disease severity, clinician judgment, and institutional practice, making confounding by indication a central concern (McCabe, 2019). Second, missingness is often informative in clinical datasets; for example, whether laboratory tests or imaging studies are performed may itself reflect clinical concern, so treating missing values as random can bias estimates or model performance (Groenwold, 2020; Madden et al., 2016). Third, errors in defining index time or exposure timing can introduce time-related biases, such as immortal time bias, and distort analyses of treatment effects or prognosis (Tyrer et al., 2022).

In comprehensive cohort databases, a common methodological problem is the overinterpretation of findings from large-scale exploratory analyses. Because these resources enable exposure-wide, phenome-wide, or multimodal screening, analyses are prone to false-positive findings and unstable results when exploratory findings are not clearly distinguished from pre-specified or independently replicated analyses.

In omics databases, several methodological problems are especially common. First, technical artifacts and sample heterogeneity are common sources of bias and interpretive uncertainty in omics analyses. Batch effects, platform differences, normalization choices, and annotation discrepancies can all introduce systematic bias, while tissue heterogeneity and cell-composition differences may further complicate interpretation, especially in bulk-data analyses (Jaffe and Irizarry, 2014; Leek et al., 2010). Second, high-dimensional omics analyses are prone to overfitting and unstable feature selection, especially when the number of measured features greatly exceeds the number of samples and findings are not adequately validated (Rahnenführer et al., 2023; Łukaszuk et al., 2024).

### 5.3 Recommended practices for rigorous and appropriate use of public biomedical databases

To improve the rigor, reproducibility, and interpretability of research using public biomedical databases, several broad principles should guide study design, analysis, and reporting.

First, researchers should select the database according to a clearly defined research question and state explicitly whether the analysis is descriptive, exploratory, predictive, or intended to support causal inference. Before finalizing database selection, investigators should determine whether the database can adequately capture the target population, key variables, timing of measurement, and major sources of confounding relevant to the question.

Second, researchers should report the database and analytic workflow transparently and in sufficient detail, including the database version, extraction date, eligibility criteria, cohort construction, variable definitions, and preprocessing steps. For studies using routinely collected health data, reporting may additionally refer to the RECORD statement (Benchimol et al., 2015). For omics studies, reporting may additionally refer to relevant minimum information reporting standards (Brazma et al., 2012).

Third, researchers should select a study design and analytic strategy that fit the research question and the structure of the available data, and should use design- and analysis-based methods to reduce bias, random error, and overinterpretation. In practice, analyses of complex survey data should incorporate sampling weights, stratification, and clustering variables to account for unequal selection probabilities and multistage sample design and to avoid biased estimates and misleading inferences (Sakshaug and West, 2014). For treatment comparisons in clinical databases, comparability between groups should be strengthened through study-design choices, such as active-comparator or new-user designs (Lund et al., 2015), and remaining confounding should then be addressed analytically, for example, with propensity score methods and, in selected settings, instrumental variable methods (Stukel et al., 2007; Austin, 2011). When missingness is plausibly informative, investigators should explicitly evaluate missing-data patterns, avoid assuming missing at random by default, use multiple imputation when appropriate, compare imputed and complete-case results, and perform sensitivity analyses under alternative missing-data assumptions (Carpenter and Smuk, 2021; Leurent et al., 2018). In high-dimensional analyses of cohort or omics data, the analytical pipeline should be prespecified, multiple testing controlled, batch effects and biological heterogeneity addressed, and exploratory analyses clearly separated from confirmatory analyses.

Finally, researchers should assess robustness through sensitivity analyses and, where feasible, independent replication or external validation, while considering generalizability and population representativeness so that conclusions do not exceed what the underlying data can reasonably support (Riley et al., 2016; Rudolph et al., 2023).

## 6 Conclusion

Public biomedical databases have become important infrastructure for contemporary biomedical research by supporting evidence generation across population, clinical, and molecular levels. Their value lies not only in improving data accessibility and supporting interdisciplinary research, but also in providing complementary evidence for surveillance, etiologic investigation, clinical prediction, and translational discovery. However, open availability does not ensure suitability, validity, or reproducibility. The scientific value of these resources depends on question-driven database selection, appropriate study design and analysis, transparent reporting, and careful assessment of robustness and generalizability. Overall, the future impact of public biomedical databases will depend not only on broader access, but also on sustained efforts to improve methodological rigor, reproducibility, and governance.
