# Supplementary material for: Resources and applications of public biomedical data
Source: Front Bioinform. 2026 May 22;6:1782956. doi: 10.3389/fbinf.2026.1782956 (PMC13236862; doi:10.3389/fbinf.2026.1782956)
Supplement: Supplementary file 1 [file Table1.docx]

Figure 1 was generated using topic-search retrieval in the Web of Science Core Collection. Searches were performed on April 23, 2026 using the names of the representative databases listed in Section 2, grouped into four categories: public health databases, clinical databases, comprehensive cohort databases, and omics databases. The search field was Topic (TS), and the retrieval was limited to English-language articles published between 2000 and 2025. Annual numbers were counted by publication year based on the retrieved Web of Science Core Collection. These counts represent records retrieved by database-name topic searches and were used to illustrate publication trends related to the listed databases. The records were not manually screened to distinguish primary data-generation studies, database descriptor or update papers, methodological papers, reviews, and secondary analyses.

Retrieval date: 2026/4/23
1. public health databases
TS=("National Health and Nutrition Examination Survey"

OR "China Health and Nutrition Survey"

OR "Korea National Health and Nutrition Examination Survey"

OR "Chinese Longitudinal Healthy Longevity Survey"

OR "Health and Retirement Study"

OR "English Longitudinal Study of Ageing"

OR "Mexican Health and Aging Study"

OR "Survey of Health Ageing and Retirement in Europe"

OR "Japanese Study of Aging and Retirement"

OR "China Health and Retirement Longitudinal Study"

OR "Midlife in the United States"

OR "Millennium Cohort Study"

OR "Global Burden of Disease"

OR "CDC WONDER" OR "Wide-ranging Online Data for Epidemiologic Research")

2. clinical databases

TS=("Surveillance, Epidemiology, and End Results"

OR "National Cancer Database"

OR "The Cancer Imaging Archive"

OR "Medical Information Mart for Intensive Care"

OR "eICU Collaborative Research Database"

OR "National Sleep Research Resource"

OR "Alzheimer's Disease Neuroimaging Initiative"

OR "Open Access Series of Imaging Studies"

OR "DeepLesion")

3. comprehensive cohort databases

TS=("UK Biobank" OR "China Kadoorie Biobank")

4. omics databases

TS=("Sequence Read Archive"

OR "European Nucleotide Archive"

OR "The Cancer Genome Atlas Program"

OR "Gene Expression Omnibus"

OR "ArrayExpress"

OR "The Encyclopedia of DNA Elements"

OR "The Human Protein Atlas"

OR "Roadmap Epigenomics"

OR "Functional ANnoTation Of the Mammalian genome"

OR "The Genotype-Tissue Expression"

OR "1000 Genomes Project"

OR "The Human Metabolome Database")
